# Knowledge of potential risk of blood-borne viral infections and tattooing practice among adults in Mandalay Region, Myanmar

**DOI:** 10.1371/journal.pone.0209853

**Published:** 2019-01-10

**Authors:** Kyaw Lwin Show, Le Le Win, Saw Saw, Chomar Kaung Myint, Kyi Maw Than, Yin Thet Nu Oo, Khin Thet Wai

**Affiliations:** 1 Department of Medical Research, Ministry of Health and Sports, Yangon, Myanmar; 2 University of Public Health, Yangon, Myanmar; Chinese University of Hong Kong, HONG KONG

## Abstract

**Introduction:**

Tattooing especially gains popularity among both men and women in adulthood from the wide range of socioeconomic groups and is noted as a risk taking behaviour in adults. Especially when tattooing does not perform to the highest standards, it can potentially be the hazardous practice. Myanmar has a paucity of evidence-based information on the estimated prevalence of tattoos and awareness of potential disease transmission from tattooing under insanitary conditions as well as the infection risk. The present research was undertaken to help identify the self-reported prevalence of tattooing among adults (18–35 years) and their knowledge of transmission risk of blood-borne infections and its determinants.

**Methods:**

A community-based cross-sectional study focused on residents aged 18–35 years was carried out in two urban and two rural areas in Mandalay district, Mandalay Region during 2015. Trained interviewers used a pre-tested structured questionnaire for face-to-face interviews with one eligible participant per selected household (n = 401). Bivariate analysis and multivariable analysis using binary logistic regression were done to ascertain the relevant explanatory variables.

**Results:**

The overall self-reported prevalence of tattooing was 19.5% (78/401) (95% CI = 16–24). Nearly 80% of participants (318/401) knew at least one blood-borne viral infection that could be transmitted from tattooing. The persons who had high formal education, manual laborers and those who lived with their families were significantly more likely to cite at least one blood-borne viral infection. Their perceived possibility to remove tattoo independently influenced the practice of tattooing (aOR = 1.91, 95% CI = 1.06–3.45; p = 0.03) compared with participants who reported no perceived possibility. Tattooing was more common in male (aOR = 13.07, 95% CI = 6.25–27.33; p<0.001) compared to female which was independently significant.

**Conclusions:**

This study ascertained the tattoo prevalence as two in ten adults of working age especially among male in central part of Myanmar in the context of lack of registration system for tattoo parlours and the issuance of safety guidelines. Findings have suggested the target groups and risk factors to be included in future health promotion programs. Future research directions should focus on perspectives of tattooists to create and sustain the sanitary practices to reduce the chance of transmission of blood-borne viral infections.

## Introduction

Globally, the practice of body art that includes skin piercing and tattooing is increasingly common in the modern society. The online survey in 2015 by The Harris Poll reported three in ten adults in the United States had a tattoo indicated the acceptability with declining negative connotations [1]. Tattooing especially gains popularity among both men and women in adulthood from the wide range of socioeconomic groups. It is noted as a risk taking behaviour in adults mainly for aesthetic reasons [2–10]. Tattooing involves piercing of the skin by the use of sharp instruments and inking the skin with one or more pigments to form a permanent design. Therefore, tattooing carries not only a risk for non-infectious conditions but also the transmission of blood-borne infections including Human Immunodeficiency Virus (HIV), Hepatitis B, Hepatitis C, septicemia, tetanus, etc. [11–14]. Tattooing should only be undertaken by the skilled, competent person with appropriate health knowledge. Especially when tattooing does not perform to the highest standards, it can potentially be the hazardous practice. In many developed countries, there are public health regulations to perform safe tattooing [15–16]. In Myanmar, dangers imposed by skin piercing instruments especially for prevention of HIV, Hepatitis B and C transmission appear in health information, education and communication materials as advocated by Disease Control Programmes under the Department of Public Health [17]. However, there are no specific local regulations concerning registration of tattoo parlours and no issuance of safe practice guidelines. In this context, Myanmar has a paucity of evidence-based information on the estimated prevalence of tattoos in adults and awareness of potential disease transmission from tattooing under insanitary conditions. Nevertheless, there is no documented evidence from Myanmar concerning tattooing as the main transmission route of blood-borne viral infections. In the light of above facts, the present research was undertaken to help identify the self-reported prevalence of tattooing among adults (18–35 years) and their knowledge of transmission risk of blood-borne infections and its determinants.

## Methods

### Study design and site

We conducted a cross-sectional descriptive study from August to December 2015 in two urban and two rural areas of Mandalay district which was included in Mandalay Region in the central part of Myanmar.

### Study population and sample size

For adults aged 18–35, we have assumed the anticipated population proportion [P] or the prevalence of self-reported tattooing as 50% due to lack of accurate estimates. With 95 percent confidence level (margin of error = 0.05) and 5 percent non-response rate, we calculated a sample size of 406.

### Sampling procedures

According to the National Census Report 2014 [18], the population of the target group (18–35 years) in the sampling frame in Mandalay district is approximately 5.1 million (urban and rural combined) residing in one million households. A multi-stage sampling procedure was used. The research team included four out of seven townships that comprised both urban and rural areas in Mandalay district with population density ranged from 445 to 9,455 inhabitants/km^2^. The remaining three townships covered downtown urban areas only. Then, two urban wards and two villages were selected purposively after discussion with Mandalay Regional Public Health Department and administrative authorities in which there were plenty of tattoo parlors. Around 100 households with working age group (18–35 years) in each study site were selected at random to meet the sample size of 406 eligible respondents. The United Nations defines youth as those persons between the ages of 15 and 24 years [19]. However, in Myanmar, the minimum age required to give consent as a competent person is 18 years. Therefore, we categorized 18–24 years as youth and 25–35 years as the older age group in this study. The gender was not considered during the selection process. The gender ratio in this study was solely resulting from the randomly recruited eligible respondents in selected households.

The list of eligible households in selected sites was provided by administrative authorities. If the eligible person was not available during the household visit at day time between 09:00 to 17:00 hours, the team replaced the nearest eligible household. There were no callbacks due to time constraint and operational feasibility. If two or more persons between 18–35 years of age were available for the interviews in the chosen household, one participant was selected randomly.

### Data collection

We trained interviewers to use a pre-tested structured questionnaire for face-to-face interviews with one eligible participant per selected household respectively. The structured interview questionnaire (SIQ) covered three components: social and demographic characteristics, knowledge and tattooing practice. In the knowledge component, there were six items: possibility of tattoo removal, methods of tattoo removal, types of needle used for tattooing, appropriate place for tattooing, adverse effects after tattooing and infectious diseases transmitted through tattooing. The interviewer noted the tattoo practice as a self-reported measure by each respondent.

### Data analysis

After checking for consistency and completeness, collected data were entered by Epi-data version 3.2 (EpiData Association, Denmark) and analyzed by IBM SPSS Statistics version 22.0 (SPSS, Inc., Chicago IL, USA). Chi-square test was used for cross-tabulation and the relative confidence intervals at 95% were calculated. A p value of <0.05 was considered statistically significant. The overall prevalence was stratified by age group and gender, and 95% CI were computed based on binomial distribution. We have chosen the response to a knowledge question “What kind of infections can be transmitted through tattooing?” as an important outcome variable 1 in this study. For this question, there are three sub items (multiple responses): knowledge of transmission of HIV infection through tattooing, knowledge of transmission of Hepatitis B virus infection through tattooing and knowledge of transmission of Hepatitis C virus infection through tattooing. Then, we transformed “Yes” response to these sub-items into one composite measure of knowledge of transmission of any one of those three blood-borne viral infections (known at least one infection). Chi squared test was computed to find out the contribution of socio-demographic characteristics on differences in knowledge of correctly identifying at least one infection that can be transmitted by tattooing not done under sanitary conditions. Either positive or negative responses to tattooing practice was considered as the second outcome variable. The binary variable was given the value of ‘1’, otherwise ‘0’. Bivariate analysis (crude odds ratio) and multivariable analysis (adjusted odds ratio) were done using binary logistic regression to ascertain the relevant explanatory variables for both outcomes. Variables with p value less than 0.2 were included as covariates to fit the final model.

#### Ethics approval and consent to participate

We obtained written informed consent from the study participants in each household and kept all information confidential. Privacy, confidentiality and anonymity issues were taken into account according to Helsinki Declaration. This study was approved by Ethics Review Committee, Department of Medical Research, Ministry of Health and Sports, Myanmar.

## Results

Four hundred and one respondents participated in the household survey (98.8%). The non-response rate in this study referred to refusals to participate in the survey. The reasons for high response rate included thorough arrangements with ward/village authorities before the survey and tattooing as a less sensitive topic for the general public compared to other individual risk behavior surveys. Among the participants, the sex ratio was 1:1.46 (69 male per 100 female) and the mean age was 25 ± 5.19 years. Approximately half (198/401) of the participants were aged between 18–24 years and 31.4% had formal education of at least high school level. Among 401 participants, 78 persons (19.5%; 95%CI = 16–24) self-reported that they had at least one tattoo and male were more likely to report tattoos than female (67/78, 41%; 95% CI = 34–49). However, the reported tattooed rate in urban areas was more or less similar to rural study sites (Table 1).

**Table 1:** Prevalence of tattooing among participants, Mandalay Region, 2015 (n = 401).

	Prevalence		95% CI
	Number	Percentage	
**Overall**	78	19.5	16–24
**Age group**			
18–24 years (n = 203)	45	22.2	17–28
25–35 years (n = 198)	33	16.7	12–22
**Gender**			
Male (n = 163)	67	41.1	34–49
Female (n = 238)	11	4.6	3–8
**Residence**			
Rural	37	18.4	13–24
Urban	41	20.5	15–26

As a specific parameter, approximately 80% of participants knew that HIV/AIDS could be transmitted through tattooing. Conversely, very few (8.7%, 35/401) noted the possible transmission of hepatitis B and C viruses. Table 2 shows the associated social and demographic factors on knowledge of at least one out of three blood-borne viral infections (HIV, Hepatitis B and C). Most of the participants (79.3%) knew at least one blood-borne viral infection (a composite measure) that could be transmitted from tattooing. The persons who had high formal education, those who were manual laborers and those who lived with their families were significantly more likely to know at least one blood-borne viral infection compared to those with no and low formal education, dependents and other job holders and those who stayed without any family members (Table 2).

**Table 2:** Factors associated with knowledge on potential risk of blood-borne viral infections through tattooing, Mandalay Region, 2015 (n = 401).

Characteristics	Known at least one infection (n = 318) No. (%)	Crude OR	95% CI	Adjusted OR	95% CI
**Overall**	318 (79.3)				
**Age**					
Between 18 and 24 years	156 (76.8)	Ref.			
Between 25 and 35 years	162 (81.8)	1.36	0.83–2.21		
**Gender**					
Female	191 (80.3)	Ref.			
Male	127 (77.9)	0.87	0.53–1.42		
**Education**					
High formal education	110 (87.3)	Ref.		Ref.	
No or low formal education	208 (75.6)	0.45	0.25–0.82*	0.57	0.30–1.08
**Occupation**					
Dependent	95 (81.9)	Ref.		Ref.	
Manual worker	110 (71.0)	0.54	0.30–0.97*	0.78	0.42–1.46
Others^1^	113 (86.9)	1.47	0.73–2.95	1.55	0.75–3.23
**Living arrangement**					
Without family	16 (51.6)	Ref.		Ref.	
With family	302 (81.6)	4.16	1.96–8.83*	3.34	1.50–7.44
**Residence**					
Rural	154 (76.6)	Ref.		Ref.	
Urban	164 (82.0)	1.39	0.86–2.26*	1.42	0.84–2.40
**Tattooed**					
No	261 (80.8)	Ref.		Ref.	
Yes	57 (73.1)	0.65	0.36–1.14*	0.62	0.34–1.12

^1^.Others = Government employee, Teacher/Officer, Own business

*Variables with p value <0.2 were selected to include in the multivariate model

More than half of the participants (61%) answered that tattoo was not removable. Among them, almost half answered the possible way of removing tattoo by laser and only a few could state other procedures such as application of hot iron plates over tattoo and surgical procedures. Inflammation and infection were the most frequent responses regarding side effects of tattooing and they also cited other possible side effects such as allergy, bleeding, itchiness and mood changes.

Table 3 analyzed factors associated with tattooing. Multivariate analysis confirmed that being a male gender (adjusted OR = 13.07, 95% CI = 6.25–27.33; p value <0.001) and those who perceived possibility to remove tattoo (adjusted OR = 1.91, 95% CI = 1.06–3.45; p value = 0.033) had significant influence on self-reported tattooing practice compared to their reference categories in the final model. However, the urban and rural residence had no influence on tattooing practice in this study (Table 3).

**Table 3:** Factors associated with tattooing practice among adults, Mandalay Region, 2015 (n = 401).

Characteristics	Tattooed (n = 78) No. (%)	Crude OR	95% CI	Adjusted OR	95% CI
**Age**					
Between 18 and 24 years	45 (22.2)	Ref.		Ref.	
Between 25 and 35 years	33 (16.7)	0.70	0.43–1.16*	0.94	0.52–1.69
**Gender**					
Female	11 (4.6)	Ref.		Ref.	
Male	67 (41.1)	14.40	7.29–28.45*	13.07	6.25–27.33
**Education**					
High formal education	22 (17.5)	Ref.			
No or low formal education	56 (20.4)	1.21	0.70–2.09		
**Occupation**					
Dependent	15 (12.9)	Ref.		Ref.	
Manual worker	36 (23.2)	2.04	1.06–3.93*	0.95	0.43–2.08
Others^1^	27 (20.8)	1.77	0.89–3.51	0.62	0.27–1.45
**Living arrangement**					
Without family	5 (16.1)	Ref.			
With family	73 (19.7)	1.28	0.48–3.44		
**Residence**					
Rural	37 (18.4)	Ref.			
Urban	41 (20.5)	1.14	0.70–1.88		
**Possibility to remove tattoo**					
No and do not know	38 (13.5)	Ref.		Ref.	
Yes	40 (33.3)	3.20	1.92–5.33*	1.91	1.06–3.45
**Side effects due to tattooing**					
Known 3–4 side effects	8 (29.6)	Ref.		Ref.	
Known 1–2 side effects	49 (21.7)	0.66	0.27–1.59	1.02	0.37–2.84
Do not know	21 (14.2)	0.39	0.15–1.01*	0.62	0.21–1.87
**Awareness of transmission of HIV, hepatitis B and C**					
At least one infection	57 (17.9)	Ref.		Ref.	
None	21 (25.3)	1.55	0.88–2.75*	1.77	0.89–3.53

^1^.Others = Government employee, Teacher/Officer, Own business

*Variables with p value <0.2 were selected to include in the multivariate model

## Discussion

This is the first study reported from Myanmar focusing on tattoo prevalence as a risk behavior among adults of working age group. To date, the indexed articles from PUB MED and other databases did not include studies from Asia that reported tattoo prevalence estimates and knowledge of potential risk of blood-borne viral infections in working age group (18–35 years). Results of the present study revealed that the prevalence of tattooing among 18–35 years of age was 20% and the majority were male. More or less similar prevalence around 19% was found in the earlier study done in United States in 2008 among 496 university and college students [20]. Nevertheless, a household survey carried out in England identified that 10% of the 10,503 adults interviewed had reported their body art practices as at least a piercing on another part of the body other than the earlobes. And 16–34 year age group accounted for 46% of overall prevalence [21]. However, online survey findings by Harris Poll in 2015 has reported a higher prevalence of 30% [1]. In contrast, the prevalence of tattooing reported by Stieger *et al*., [22] from the survey in central Europe among 440 participants found only 15.2%, which was lower than the current study. A computer assisted telephone interview survey in a larger sample of 8,656 men and women in Australia [9], revealed approximately 15% had ever getting tattooed. Even though different surveys used different data collection methods, the reported prevalence of tattooing did not vary widely. However, one study which highlighted the 85% sensitivity for self-reported behaviours including tattooing stressed on the careful choice of data source depending on the type of information sought [23] to avoid measurement error/bias. Age and gender specific prevalence reported in this study identified the preponderance of younger age group (18–24 years) and male. Findings were in line with other studies from United States, Europe and Australia [20, 22, 9]. A recent qualitative study from India [24] provided the evidences of tattooing practice influenced by culture, fashion and social media among others. Henceforth, there is a globalization in tattooing practice which gains popularity in young adults that leads to an increasing trend and Myanmar is no exception.

In this study, it was apparent that the specific knowledge of likely transmission of HIV infection through tattooing was almost 10 times higher than the transmission of hepatitis B and C infections. In a survey of 103 undergraduate students from the United States, Schorzman and colleagues [25] found a high level of awareness and overestimation in the potential health risks of body art. However, Cegolon et al., [4] in 2010 reported lesser knowledge of body art related infectious diseases and mandatory hygienic rules to be observed in a larger sample of 4,277 Italian secondary school adolescents. Again when the findings were confined to outcome 1 (a composite measure): know at least one blood-borne viral infection, a significant proportion of adults could provide positive responses. This finding suggests the necessity to convey adequate health information to possible consumers on risks of blood-borne viral infections in the absence of guidelines and regulations to control unsafe body art practices. It is well documented that unsterile needles and equipment can transmit hepatitis B and C viruses [26]. In this study, age and gender did not reveal any significant statistical differences to knowledge outcome 1 as well as for tattooing practice. This finding concurred earlier studies from Europe in which male and female university students did not uncover any significant difference in tattooing [27]. As might be expected, those with higher level of formal education were significantly more likely to express the correct response compared to those without and this finding was consistent to other studies. [4, 27] However, manual laborers had less knowledge compared to dependents and other work categories. The underlying reason is unclear but unstable work conditions, prolonged working hours, and poor chance for an exposure to adequate health information might play an important role. Family and social networks favored the spread of health information [28] and this fact was evident in this study. Those who lived with their families were more likely to express correct knowledge compared to those without.

The laws, statutes and regulations to control untoward consequences of tattooing vary in certain developed countries [16]. In this study, most of the male favoured tattooing. Also, those who had knowledge of possible removal of a tattoo and those who were naive in consequences of tattooing reported the practice being proved by a well fitted multivariate model. One study from Naples, Italy [27] proved the significant association between the use of sterile instruments and the higher awareness of transmission of infections. In the absence of legislations, there should be an increased alertness amongst health care providers concerning risk of transmission of blood-borne diseases, as well as the importance of stressing to potential clients who may undertake this risk taking behavior. Translation of scientific evidences and strengthening knowledge transfer mechanisms of correct information to reach the general population required attention. Conversely, concerted efforts to engage stakeholders are essential to introduce cost-effective prevention strategies to mitigate the risk of spread of blood-borne viral infections in a developing country like Myanmar.

### Limitations of the study

This study has limitations. First, we were unable to follow the stratified cluster sampling procedure by recruiting the equal number of adult men and women in selected wards and villages. As a result, the flaw noted during the household survey was the proportion of women in the sample exceeded men due to household visits during working hours. Second, the use of a single knowledge item as first outcome variable hampered the interpretation of findings based on the knowledge score which is a composite measure of all knowledge items. Third, the study put emphasis only on potential consumers of tattooing and thus, there is a missing information on tattooing practices among professional tattooists and their knowledge of blood-borne virus infection risk associated with tattooing. Finally, in the current data set, the perspectives of healthcare providers concerning risk of transmission of blood-borne diseases to potential clients could not be included.

## Conclusions

This study ascertained the tattoo prevalence as two in ten adults of working age in central part of Myanmar in the context of lack of registration system for tattoo parlours and the issuance of safety guidelines. Being an urban dweller or a rural resident did not differ in infection related knowledge and reported tattooing practice in this study due to lack of well-informed health promotion materials, activities and proper legislations. Apart from the gap in knowledge related to tattoos and real practice, findings have suggested the target groups and risk factors to be included in future health promotion programs in Myanmar. Moreover, future research directions should focus on perspectives of tattooists to create and sustain the sanitary practices to reduce the chance of transmission of blood-borne viral infections as well as other bacterial and fungal infections directly through the skin by tattooing, or indirectly through the tattoo parlour environment.

## Supporting information

S1 File Knowledge of potential risk of blood-borne viral infections and tattooing practice Interview-administered questionnaire. (PDF)

S2 File De-identified, anonymized minimally available dataset. (SAV)

## Abbreviations

CI: Confidence Interval
HIV: Human Immunodeficiency Virus
OR: Odds Ratio
SIQ: Structured Interview Questionnaire
