# Supplementary material for: Knowledge of potential risk of blood-borne viral infections and tattooing practice among adults in Mandalay Region, Myanmar
Source: PLoS One. 2019 Jan 10;14(1):e0209853. doi: 10.1371/journal.pone.0209853 (PMC6328096; doi:10.1371/journal.pone.0209853)
Supplement: S1 File — (PDF) [file pone.0209853.s001.pdf]

Assessing knowledge and practice on health issues of tattooing among community in Mandalay Region

Start Time_______

**Ward____________**	**Township________**	**ID__________**	**Interviewer____________**

1	Age (Completed age)	|__|__|	
2	1. Male<br>2. Female	|__|	
3	Religion<br>1. Buddhist<br>2. Christian<br>3. Islam<br>4. Hindu<br>5. Others========	|__|	
4	Education<br>1. Illiterate (Can't read and write)<br>2. Can read and write<br>3. Primary school<br>4. Middle school<br>5. High school<br>6. University student<br>7. Graduate	|__|	
5	Marital status<br>1. Married<br>2. Single<br>3. Separated<br>4. Divorced<br>5. Widowed	|__|	
6	Occupation<br>================	|__|	
7	Living with<br>1. Parents<br>2. Guardians<br>3. With my own family<br>4. Self/Alone<br>5. Others____________	|__|	
Knowledge questions			
8	Do you know skin piercings including tattooing?<br>1. Yes<br>2. No	|__|	
9	Is it possible to remove tattoos after tattooing?		

<table>
<tr><td></td><td>(If answer 2 or 3, please skip to Question 11)
1. Yes
2. No
3. Don’t know</td><td>|__|</td><td></td></tr>
<tr><td>10</td><td>What are the methods of removing tattoos?
(Multiple responses)
1. Surgical procedure
2. Applying laser
3. Washing with water
4. Don’t know
5. Others______________</td><td>1.|__|
2.|__|
3.|__|
4.|__|
5.|__|</td><td></td></tr>
<tr><td>11</td><td>Is it possible to suffer some adverse effects in our bodies after tattooing?
(If answer 2 or 3, please skip to Question 13)
1. Yes
2. No
3. Don’t know</td><td>|__|</td><td></td></tr>
<tr><td>12</td><td>What kind of adverse effects can occur after tattooing?
1. Inflammation
2. Allergy
3. Bleeding
4. Itchiness
5. Infectious diseases
6. Others_______________</td><td>Not probe
1.|__|
2.|__|
3.|__|
4.|__|
5.|__|
6.|__|</td><td></td></tr>
<tr><td>13</td><td>Is it possible to transmit infectious diseases through tattooing?
(If answer 2 or 3, please skip to Question 15)
1. Yes
2. No
3. Don’t know</td><td>|__|</td><td></td></tr>
<tr><td>14</td><td>What kind of infectious diseases can be transmitted through tattooing?
1. HIV/AIDS
2. Hepatitis B
3. Hepatitis C
4. Others______________</td><td>Not probe
1.|__|
2.|__|
3.|__|
4.|__|</td><td></td></tr>
<tr><td colspan="4">Practice questions</td></tr>
<tr><td>15</td><td>Do you have tattoos?
1. Yes
2. No</td><td>|__|</td><td></td></tr>
<tr><td>16</td><td>How many tattoos do you have?</td><td></td><td></td></tr>
</table>

<table>
<tr><td></td><td>1. One
2. Two
3. Three
4. Four or more than four___________</td><td>|__|</td><td></td></tr>
<tr><td>17</td><td>Who encourage you for tattooing?
(Multiple responses)
1. Self decision
2. Friends
3. Siblings
4. Others_____________</td><td>1.|__|
2.|__|
3.|__|
4.|__|</td><td></td></tr>
<tr><td>18</td><td>What were the main reasons for tattooing?
(Multiple responses)
1. For aesthetic reason
2. To be different from others
3. To be popular
4. Because of encouraging from others
5. I don’t want to tell
6. Others_____________</td><td>1.|__|
2.|__|
3.|__|
4.|__|
5.|__|
6.|__|</td><td></td></tr>
<tr><td>19</td><td>Where did you get tattooing?
(Multiple responses)
1. Home shop
2. Tattoo shop from supermarkets
3. Tattoo shop from festivals
4. Piercers’ home
5. Others_____________</td><td>1.|__|
2.|__|
3.|__|
4.|__|
5.|__|</td><td></td></tr>
<tr><td>20</td><td>Where was the main place to get tattoos?
(Single response)
1. Home shop
2. Tattoo shop from supermarkets
3. Tattoo shop from festivals
4. Piercers’ home
5. Others_____________</td><td>|__|</td><td></td></tr>
<tr><td>21</td><td>Why did you get tattoos at that place?
(Refer to Question 20)
================================</td><td>|__|</td><td></td></tr>
<tr><td>22</td><td>Who pierce you?
(Multiple responses)
1. Professional piercer
2. Friend
3. Others____________</td><td>1.|__|
2.|__|
3.|__|</td><td></td></tr>
<tr><td>23</td><td>Who was the main person that pierces you?
(Single response)</td><td>|__|</td><td></td></tr>
</table>

	1. Professional piercer<br>2. Friend<br>3. Others____________		
24	Did you have experience on side effects of tattooing?<br>1. Yes<br>2. No	|__|	
25	If yes, what side effects did you suffer?<br>(Multiple responses)<br>1. Inflammation<br>2. Allergy<br>3. Bleeding<br>4. Itchiness<br>5. Infection<br>6. Others____________	1.|__| 2.|__| 3.|__| 4.|__| 5.|__| 6.|__|	
26	Whom did you consult/treat with?<br>1. I did not treat it<br>2. Practitioners<br>3. Medical professionals<br>4. Others____________	|__|	

End time=======================

Date ==========================
